# Trasplante autólogo de progenitores hematopoyéticos en paciente con enfermedad de Crohn refractaria

**DOI:** 10.23938/ASSN.1054

**Published:** 2023-11-15

**Authors:** Ana Gordo Ortega, Maren Eizaguirre Ubegun, Martín Balerdi Trébol, Saioa Rubio Iturria, Cristina Rodríguez Gutiérrez

**Affiliations:** Servicio Navarro de Salud-Osasunbidea Servicio Navarro de Salud-Osasunbidea Hospital Universitario de Navarra Servicio de Aparato Digestivo Pamplona Spain

**Keywords:** Enfermedad de Crohn, Trasplante autólogo, Células madre hematopoyéticas, Médula ósea, Crohn disease, Transplantation, autologous, Hematopoietic stem cell, Bone marrow

## Abstract

La enfermedad de Crohn es una condición crónica para la que en ocasiones no existe tratamiento efectivo, ni médico ni quirúrgico. En estos casos, el trasplante autólogo de progenitores hematopoyéticos puede ser una opción terapéutica con la que restaurar la tolerancia inmunológica del paciente. En algunos casos se conseguirá la remisión de la enfermedad o un descenso en su actividad, haciendo que fármacos que habían fracasado vuelvan a ser efectivos. El perfil de seguridad del procedimiento, unido al hecho de que no es un tratamiento curativo, hace que la selección de los pacientes tenga que ser muy rigurosa.

Presentamos nuestra experiencia con el primer paciente seleccionado en nuestro centro para trasplante autólogo de progenitores hematopoyéticos: un varón de 27 años con enfermedad de Crohn (A1L3B1p) refractaria a múltiples líneas de tratamiento médico y no candidato a tratamiento quirúrgico, que dos años tras el trasplante se encuentra asintomático.

## Abstract (en)

Crohn’s disease is a chronic condition for which sometimes there is no effective medical or surgical treatment. Autologous hematopoietic stem cell transplantation may be a therapeutic option for these patients to restore immune tolerance. Consequently, this may lead to remission of the disease or decrease its activity, making drugs that have previously failed be effective. Due to the safety profile of the procedure and the fact that it is a non-curative treatment, patient selection must be rigorous.

We report our experience with the first patient selected in our centre for autologous hematopoietic stem cell transplantation: 27 years old male with Crohn’s disease (A1L3B1p) refractory to multiple lines of medical treatment and not a candidate for surgical treatment. Two years after the transplantation, the patient remains asymptomatic.

## INTRODUCCIÓN

La enfermedad de Crohn (EC) se caracteriza por la presencia de inflamación en cualquier parte del tubo digestivo. Su evolución es impredecible, siendo lo más habitual el curso crónico y recidivante, alternando épocas de actividad con otras en las que la enfermedad se mantiene inactiva^1^. En Navarra, su incidencia se estima en 7,1 casos/10^5^ habitantes/año, similar a la media nacional, de 7,5 casos/10^5^ habitantes/año^2^.

Aunque en las últimas décadas ha habido grandes avances terapéuticos en la EC, en ocasiones no se encuentra un tratamiento efectivo, ni médico ni quirúrgico. En estos casos, el trasplante de progenitores hematopoyéticos (TPH) autólogo puede ser una opción a valorar^3^. Presentamos el primer caso de un trasplante de TPH realizado a un paciente con una EC multirrefractaria, con objetivo de dar a conocer esta opción terapéutica.

## CASO CLÍNICO

Paciente varón de 27 años, con antecedentes de psoriasis y nefropatía IgA, que debutó a la edad de 16 años con una EC ileocolónica y perianal (A1L3B1p). Durante los primeros 10 años de evolución la enfermedad se mostró refractaria a múltiples líneas de tratamiento, incluyendo fármacos clásicos como la azatioprina, o biológicos como los anti-TNF, vedolizumab combinado con metrotrexate, o ustekinumab. Ante la falta de respuesta, se usó de manera compasiva certolizumab, sin éxito, por lo que el paciente fue incluido en un ensayo clínico con upadacitinib, con el que tampoco se obtuvo respuesta.

Ante el fracaso terapéutico se realizó una ileostomía derivativa con idea de excluir el tránsito cólico (que era la zona con mayor actividad) para favorecer la cicatrización mucosa, y se reanudó el tratamiento con infliximab, que había sido la molécula más efectiva en este paciente, aunque sin llegar a conseguir remisión. Esta estrategia también resultó ineficaz.

Dada la localización de la enfermedad en el paciente, el tratamiento quirúrgico implicaba una ostomía permanente, por lo que se valoró el TPH autólogo como siguiente estrategia. El paciente fue derivado al Hospital Clìnic de Barcelona (España), centro con experiencia en esta indicación de trasplante y donde se llevó a cabo el proceso de selección.

Este proceso radica en la necesidad de asegurar que el paciente está en las mejores condiciones para enfrentar un procedimiento no exento de efectos adversos graves (incluyendo la muerte), principalmente por eventos cardiovasculares e infecciones. Para ello se realizó una evaluación cardiorrespiratoria con pruebas funcionales y de imagen, un despistaje de focos de infección, un aspirado de médula ósea y una prevención de otros potenciales efectos adversos como la pérdida de masa ósea y la infertilidad.

Además, se reevaluó el estado de la enfermedad con una colonoscopia, y se realizó una resonancia magnética nuclear (RMN) abdominal para descartar formas de enfermedad no susceptibles de mejoría, como son aquellas con componente fibrótico, o enfermedad perianal activa, la cual no solo no responde al trasplante, sino que se relaciona con mayor tasa de recaídas y complicaciones^4^^,^^5^.

El TPH se llevó a cabo en tres fases. Se obtuvieron las células progenitoras hematopoyéticas mediante aféresis tras su movilización a la sangre periférica con factor estimulante de colonias de granulocitos. A continuación, tuvo lugar el proceso de acondicionamiento mieloablativo, para lo cual se administró ciclofosfamida (50 mg/Kg/día durante 4 días) y globulina antitimocítica (2,5 mg/Kg/día durante 3 días). Finalmente, se llevó cabo la infusión de los progenitores hematopoyéticos que habían sido criopreservados tras la aféresis.

Durante el ingreso en el centro trasplantador, el paciente presentó complicaciones post TPH como fiebre neutropénica y derrame pericárdico, que se resolvieron sin incidencias. También recibió el régimen de profilaxis infecciosa necesario.

Posteriormente, y ya en nuestro centro, el paciente recibió todas las vacunas que le habían sido administradas previamente a lo largo de su vida, dado que un TPH supone también la perdida de la memoria inmunológica^5^.

A los seis meses post TPH, el paciente se encontraba en remisión sin necesidad de tratamiento de mantenimiento, por lo que se reconstruyó el tránsito intestinal y se pautó tratamiento preventivo con vedolizumab cada ocho semanas.

La presencia de actividad inflamatoria en una colonoscopia de control realizada doce meses post TPH obligó a intensificar el tratamiento con vedolizumab, administrándose cada cuatro semanas; se documentó respuesta en los dos meses posteriores.

Actualmente, 22 meses post TPH, el paciente se mantiene en tratamiento con vedolizumab cada cuatro semanas, y se encuentra en seguimiento en su centro por los servicios de Hematología y Aparato Digestivo, y por el servicio de Digestivo del centro trasplantador, encontrándose a día de hoy asintomático.

## DISCUSIÓN

La enfermedad de Crohn representa la tercera indicación de TPH por patología autoinmune por detrás de la esclerosis múltiple y de las enfermedades del tejido conectivo. España es el país con más trasplantes en esta indicación, con 66 casos registrados a fecha de febrero de 2022^6^

El mecanismo por el que el TPH logra controlar la enfermedad no es bien conocido, pero se cree que puede restaurar la tolerancia inmunológica mediante la erradicación de las respuestas inmunitarias patológicas y la reconfiguración del sistema inmunológico^4^^,^^3^. Aunque el TPH autólogo no logra modificar la susceptibilidad genética sujeta al desarrollo de la enfermedad, el TPH alogénico, que podría tener un mayor efecto curativo, no se contempla como tratamiento dado que sus efectos adversos y la tasa de mortalidad no se consideran aceptables para el tratamiento de esta enfermedad crónica autoinmune^4^^,^^7^.

Algunos estudios previamente publicados han demostrado que el TPH supone una mejoría en los índices de actividad tanto clínicos como endoscópicos, lo que en práctica clínica se traduce en que más de la mitad de los pacientes responden a tratamientos a los que antes habían fracasado, o que incluso en algunos casos se pueda prescindir del tratamiento de mantenimiento^8^^,^^9^.

En definitiva, el TPH autólogo es una opción en pacientes refractarios o no candidatos a tratamiento médico o quirúrgico. El perfil de seguridad del procedimiento, unido al hecho de que no supone un tratamiento curativo y a que hay formas de enfermedad no susceptibles de mejoría, hace que la selección de los pacientes deba ser muy rigurosa. A pesar de que en ocasiones no se consigue la remisión de la enfermedad, sí puede modificarse su actividad, de manera que tratamientos que previamente habían fracasado pueden convertirse en fármacos efectivos, como es el caso de nuestro paciente.
